# Biomass and coccolith productions in *Chrysotila* sp. in high density cultivation system

**DOI:** 10.1007/s00253-026-13907-w

**Published:** 2026-06-10

**Authors:** Matteo Ferrari, Veronica Buson, Elisa Palandri, Manuela Bordiga, Adriano Carrara, Eleonora Sforza

**Affiliations:** 1 Department of Industrial Engineering, University of Padua, Via Marzolo 9, 35131 Padua, Italy; 2 National Institute of Oceanography and Applied Geophysics (OGS), Borgo Grotta Gigante 42/C, 34010 Sgonico, Trieste, Italy; 3 A2A S.p.A., Brescia, Italy

**Keywords:** Coccolithophores, Coccolith, Semicontinuous cultivation, PIC:POC, Biomass production

## Abstract

**Abstract:**

Coccolithophores are unicellular marine microalgae capable of producing calcium carbonate exoskeletons composed of micrometric scales called coccoliths. Since they require CO₂ for both calcification and biomass production, they are expected to exhibit a potentially higher CO₂ fixation, making them promising candidates for applications in biological carbon capture systems. In addition, coccoliths themselves are considered high-value products due to their potential applications in many fields such as nanobiotechnology. In this study, a high cell density culture of *Chrysotila* sp. was tested using the CellDEG^®^ technology, with the objective of maximizing both biomass and coccolith production as a function of light intensity, nitrogen and carbon supply, thanks to a design of experiments (DoE) approach. The optimal conditions identified for biomass production were 600 mg L⁻^1^ of nitrogen and a light intensity of 375 µmol m⁻^2^ s⁻^1^, which allowed the system to reach a final biomass productivity of 0.96 g L⁻^1^ d⁻^1^. However, coccolith production was found to be very limited under these conditions. Additional experiments assessed the effect of calcium addition and light regime, which did not remarkably affect the PIC:POC ratio. On the other hand, when *Chrysotila* sp. was cultivated in a semicontinuous system with controlled pH (to maintain the carbonate structure of the coccosphere) with a light bubbling as mixing, a remarkable particulate inorganic carbon versus particulate organic carbon ratio (PIC:POC) of 0.3 was obtained. Semicontinuous feeding was selected for its ability to maintain relatively stable concentrations of nutrients and cells over time. Therefore, semicontinuous cultivation under these conditions is recommended for coccolith production, whereas the CellDEG^®^ system is more suitable for biomass generation. This study allowed us to obtain data on the growth of *Chrysotila* sp. using semicontinuous and high cell density culture systems, which is innovative given the limited information available in literature regarding the cultivation of this species.

**Key points:**

- *CellDEG*^®^* technology allows high biomass production, but low PIC:POC*
- *Changes in calcium concentration or photoperiod cannot increase CellDEG*^®^*’s PIC:POC*
- *Semicontinuous cultivation yields high PIC:POC but reduces biomass levels*

**Graphical Abstract:**

**Figure:**
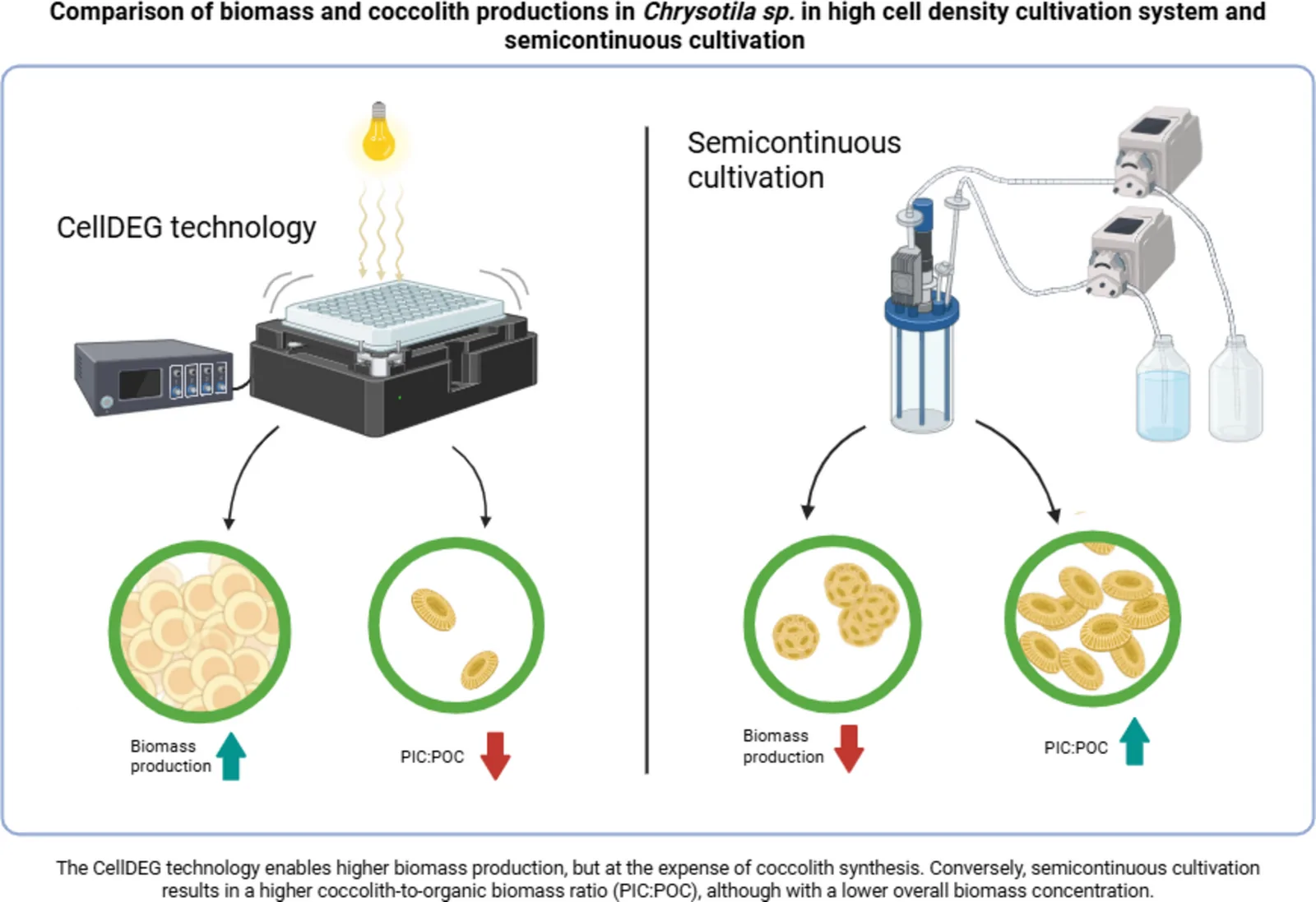

**Supplementary Information:**

The online version contains supplementary material available at 10.1007/s00253-026-13907-w.

## Introduction

Carbon dioxide (CO₂) is the main greenhouse gas produced by human activities (Bai and Yeh 1997; Lee et al. 2023), and despite international regulations (Wolf et al. 2021), its atmospheric concentration continues to rise, posing serious risks to ecosystems and human well-being (Bhola et al. 2014; Nunes 2023). According to the Global Energy Review (IEA 2025), global CO₂ emissions from energy combustion and industrial processes reached 37.8 Gt in 2024, representing a 0.8% increase compared to 2023.This global challenge has intensified the need for strategies capable of reducing CO₂ emissions. There are three main approaches to CO₂ sequestration: physical, chemical, and biological (such as microalgae-based systems) (Zhou et al. 2017). Among the proposed solutions, the use of microorganisms to capture CO₂ from flue gases is gaining increasing attention (Dębowski et al. 2020), since physical methods are associated with low long-term storage stability and potential environmental risks, whereas chemical methods require high-purity CO₂, which is typically not available in industrial flue gases, expensive chemical materials such as solvents, adsorbents, and membranes, and high energy consumption (Liu et al. 2023; Wang et al. 2025; Xie et al. 2024). On the other hand, bio-based carbon capture systems exhibit lower energy consumption and fewer transportation and leakage issues compared to conventional carbon capture technologies based on physical and chemical methods (Onyeaka et al. 2021). Among the microorganisms employed in biological approaches for CO₂ capture are methanotrophic bacteria and microalgae. While some methanotrophic bacteria are capable of enzymatically converting CO₂ into reduced carbon compounds such as methanol in the presence of excess CO₂ (Sahoo et al. 2021), microalgae rely on CO₂ as their primary carbon source and convert it into cellular biomass (Ma et al. 2022; Sun et al. 2025). Within this context, the latter represent a particularly promising option: they exhibit a carbon capture capacity up to 40 times greater than that of terrestrial plants and are not constrained by geographical or agricultural limitations (Nguyen et al. 2023; Sen and Mukherjee 2024). In addition, their ability to metabolize toxic by-products such as nitrogen oxides, together with their physiological versatility and resilience to the extreme conditions of flue gases, enables them to capture CO₂ directly from point-source emissions (Li et al. 2023; Thomas et al. 2016; Wang et al. 2025). In particular, coccolithophores, since they produce external calcite scales known as coccoliths, enabling them to permanently fix inorganic carbon (Balch 2018; Rickaby et al. 2016; Young 1997). Productivities of 0.56 g L⁻^1^ d⁻^1^ and 0.17 g L⁻^1^ d⁻^1^ were previously reported by Moheimani et al. (2011) and Dolganyuk et al. (2023) obtained with *C. carterae*, showing preliminary data that are promising. Their unique ability to biomineralize calcium carbonate also highlights their potential in nanotechnology. Their distinct morphology has been linked to promising biomedical applications, and, importantly, coccoliths are not essential for coccolithophore survival (Lomora et al. 2019; Walsh et al. 2018). This makes their structure and production especially amenable to bioengineering, generating opportunities for high-value biogenic nanomaterials that could support the economic viability of industrial cultivation systems (Skeffington and Scheffel 2018). In CO₂ capture processes, increasing biomass concentration is crucial to maximize uptake. While conventional photoautotrophic systems typically reach around 1 g L⁻^1^ of dry biomass (Lippi et al. 2018), high cell density cultures (HCDC) can exceed 10 g L⁻^1^ under optimal conditions, depending on species and cultivation technology (Bähr et al. 2016; Lucato et al. 2025; Tizzani et al. 2025; Zhou et al. 2024). However, HCDC applications for coccolithophores remain scarcely explored. Optimizing such systems requires defining key operational parameters, including temperature, pH, light, nutrients, aeration, and mixing (Razzak et al. 2023; Yusoff et al. 2019). Traditional one-factor-at-a-time experimentation is slow and inefficient for identifying optimal growth conditions (Gassenmeier et al. 2022). In contrast, design of experiments (DoE) methodologies allow simultaneous evaluation of multiple variables, increasing experimental efficiency, reducing costs, and identifying interactions that would otherwise remain undetected (Barrentine 2001; Ilzarbe et al. 2008; Jankovic et al. 2021). This study aims at optimizing biomass and coccolith production in *Chrysotila* sp. with a DoE approach assessing the effects of light intensity, CO₂ supply, and nitrogen concentration on growth under high-density conditions. In addition, the effect of different cultivation modes, including semicontinuous systems, were assessed. Additional experiments investigated the influence of photoperiods and calcium availability on coccolith formation. The resulting data provide a detailed characterization of *Chrysotila* sp. physiology in industrially. The novelty of the paper relies on the application of high-density cultivation systems on coccolithophores, showing the highest biomass productivity reported for this taxonomical group. In addition, this study investigates the effect of cultivation conditions on the quantitative production of coccoliths.

## Materials and methods

### Coccolithophore strain and culture conditions

The species employed in this study is *Chrysotila* sp., a golden-brown microalga belonging to the coccolithophore group, obtained from the Roscoff Culture Collection (RCC). The culture was maintained in a 500-mL flask filled with a modified f/2-based medium.

The medium is prepared starting from deionized water and adding 30 g L^−1^ of *Tropic Marin® Classic* sea. The water is then autoclaved and enriched with microfiltrated (pore size 0.22 µm) stock solutions of macronutrients, trace elements and vitamins to achieve the following final concentrations in the culture medium: 0.425 g L^−1^ of NaNO_3_, 0.031 g L^−1^ of NaH_2_PO_4_, 0.0043 g L^−1^ of Na_2_EDTA*2H_2_O, 0.0032 g L^−1^ of FeCl_3_*6H_2_O, 6.29E-06 g L^−1^ of Na_2_MoO_4_*2H_2_O, 2.2E-05 g L^−1^ of ZnSO_4_*7H_2_O, 9.99E-06 g L^−1^ of CoCl_2_*6H_2_O, 0.00018 g L^−1^ of MnCl_2_*4H_2_O, 9.81E-06 g L^−1^ of CuSO_4_*5H_2_0, 1.15E-06 g L^−1^ of H_2_SeO_3_, 1.08E-06 g L^−1^ of NaVO_3_, 2.38E-06 g L^−1^ of NiSO_4_*6H_2_O, 0.0001 g L^−1^ of thiamine-HCl, 5.2E-08 g L^−1^ of biotin and 5.55E-07 g L^−1^ of vitamin B12 (Palandri et al. 2026). To maintain a stable pH of 8.5 during cultivation, Trizma buffer was added to the medium after UV sterilization to reach a final concentration of 40 mM. From Table 1, it is evident that particular attention was given to providing high concentrations of nitrogen and phosphorous, to avoid nutrient-limiting conditions during cultivation. The cultures were continuously exposed to white LED light at an intensity of 100 µmol m⁻^2^ s⁻^1^, and internal mixing was ensured through bubbling. This aeration system also provided effective oxygen stripping from the culture medium.

**Table 1:** Experimental plan generated by the design of experiment for the CellDEG^®^ experiments

Condition	N (mg L^−1^)	I (µmol m^−2^ s^−1^)	Number of replicates
1	150	600	× 1
2	150	375	× 3
3	150	150	× 1
4	375	600	× 3
5	375	375	× 6
6	375	150	× 3
7	600	600	× 1
8	600	375	× 3
9	600	150	× 1

### High cell density cultivation: CellDeG^®^

For high-density cultivation, the patented CellDEG^®^ system (CellDEG^®^ GmbH, Germany, patent number 9200244) was employed. This system provides two types of photobioreactors: 100 mL (HD100) and 10 mL (HD10) units. These can be mounted on orbital shaking platforms (HDC 9.100 for the 100 mL reactors and HDC 45.10 for the 10 mL reactors), which ensure constant mixing at 110 rpm and enable air supply into the photobioreactors. Air is delivered through nanoporous hydrophobic membranes located at the base of the photobioreactors, allowing bubble-free aeration. In addition, CO₂ delivery can be precisely controlled via a growth control unit (GCU) connected to the platform. Illumination is provided from above by a perpendicular white light source (LED KE 308, DH Licht GmbH, Germany). For further details on the system, refer to the comprehensive diagram provided by Lippi et al. (2018). Light intensity and photoperiod are set depending on the experiment, as described in the following sections,

#### DoE on nitrogen, light intensity and CO_2_%

In order to optimize the growth of *Chrysotila* sp. in a high-density cultivation system such as the CellDEG^®^ platform, it was necessary to test different growth conditions, including light intensity, nitrogen concentration in the medium and the amount of supplied CO₂. Since this system allows for a large number of simultaneous experiments (up to 33), a design of experiments (DoE) approach was considered the most efficient way to evaluate these parameters in an optimization exercise on the biomass growth. The independent variables were nitrogen concentration (three levels), CO_2_ partial pressure (two levels) and light intensity (three levels) while the dependent responses were biomass productivity and PIC:POC ratio. The response surface methodology (RSM) was employed within this framework, using a full factorial central composite design. The input variables were selected in order to fill the knowledge gap about this species in a quantitative way. Upon collection of the experimental results, a mathematical model for each response was developed through statistical analysis. The selected mathematical models underwent a reduction process: only the significant factors, including possible interactions and factor dependencies, exhibiting a p-value lower than 0.05, were kept in each model. As highlighted by experiment frequency, some experimental conditions were tested in replicates: the central and the axial points of the design space were replicated. Replicates at the central point allowed the estimation of the pure error, which was not due to the input factors but to the experimental intrinsic variability. Once the pure error was defined, the software calculated the lack-of-fit, which is a statistical parameter representing the goodness of the mathematical model in fitting the experimental data set. The model reliability is represented by a statistically non-significant lack-of-fit value (*p*-value > 0.1) and by a difference between the adjusted and predicted *R*^2^ lower than 0.2. The adjusted *R*^2^ is an index of the amount of variation in the data set that is explained by the model. On the other side, the predicted *R*^2^ reflects the amount of variation in prediction that is explained by the model. The latter should be within 0.2 of the adjusted *R*^2^ to avoid model over-fitting. Multiple regression analysis of variance was applied to evaluate the significance of different-order polynomial equations derived from the experimental data. For each response, the mathematical model that best described the effect of the input variables was selected, based on a p-value lower than 0.5 and an adjusted *R*^2^ value indicating a good fit (*R*^2^ > 0.7). Nonlinearity was checked and the corresponding results are commented in the results section. The Design-Expert software was used to generate the experimental plan (Table 1) applied with 1% and 10% CO_2_. The experimental factors evaluated were light intensity (I) with three levels (150, 375, and 600 µmol m⁻^2^ s⁻^1^) and nitrogen concentration (N) with three levels (150, 375, and 600 mg L⁻^1^), while the dependent variables were biomass productivity ($P{m}_{tot}$, g L^−1^ d^−1^) and particulate inorganic carbon versus particulate organic carbon ratio (PIC:POC). The experiment was also repeated using two different CO₂ concentrations in the inlet air (1% and 10% v/v). Regarding CO2 supply, a CO₂ level of 1% was chosen because it is commonly reported as sufficient and non-limiting for microalgae growth (Taher et al. 2015). Conversely, 10% CO₂ was selected as it represents a typical average concentration found in flue gas streams (Chapel et al. 1999; Wang and Song 2020). It is important to specify that, each time the medium composition was modified to increase the nitrogen concentration, the phosphorus concentration was also increased in order to maintain a molar N:P ratio of 19:1.

Each experiment was conducted for one week using an f/2 medium (modified in nitrogen and phosphate according to the experimental plan) under continuous illumination (24 h of light). The experiment was carried out using the HD10 vessel as the photobioreactor. Biomass productivity ($P{m}_{tot}$) and PIC:POC ratio were evaluated as output responses. Upon collection of the experimental data, the mathematical model was developed for each response through statistical analysis and details are discussed in the results section. The reduced mathematical model was validated according to the conditions suggested by the software. Validation experiments were performed in duplicate.

#### CellDEG^®^ experiments: photoperiod of 18:6 h light–dark

Following the results obtained from the experiment conducted using the Design of Experiment (DoE) approach, a subsequent set of experiments was carried out using the CellDEG^®^ system, without adopting a DoE approach. In this experiment, the culture was exposed to a photoperiod of 18:6 h light–dark, differently from the DoE-based experiment where continuous illumination was applied. This variation was introduced to assess whether changes in the photoperiod could influence coccolith synthesis. This experiment was conducted by testing different conditions of light intensity and nitrogen concentration (Table 2), while maintaining the CO₂ concentration at 1%. This concentration was selected because it yielded better results in terms of the PIC:POC ratio in the previous experiment. The remaining settings were kept identical to those used in the CellDEG^®^ experiment conducted using the DoE approach, and the entire experiment lasted 1 week.

**Table 2:** Experimental plan for the CellDEG^®^ experiment with calcium supplementation

Condition	N (mg L^−1^)	I (µmol m^−2^ s^−1^)	Number of replicates
1	150	150	X2
2	150	375	X2
3	150	600	X2
4	375	150	X2
5	375	375	X2
6	375	600	X2
7	600	150	X2
8	600	375	X2
9	600	600	X2

In this experiment as well, biomass productivity and the PIC:POC ratio were measured.

A further CellDEG^®^ set of experiments (not under DoE approach) was conducted to assess whether a calcium supplement would promote the formation of a calcium carbonate shell. To this end, various concentrations of calcium chloride were added to the culture medium. The concentration of other salts was adjusted accordingly to maintain the original ionic concentration of the medium. The tested calcium chloride concentrations were 3, 6, and 9 g L^−1^, along with a control condition without added calcium chloride. Each of these calcium conditions was tested at two nitrogen levels: 600 and 850 mg L^−1^. The light intensity was set at 375 µmol m⁻^2^ s⁻^1^, the cultures were aerated with air enriched with 1% CO₂ and the total duration of the experiment was 1 week.

### Semicontinuous cultivation

To investigate the effects of CO₂ and pH reduction on the formation of the coccosphere, a semicontinuous cultivation experiment was carried out, with pH regulation controlled by a sensor. In this setup, the pH is maintained around 8.5 (an optimal range for preserving the carbonate of the coccolith) by supplying CO₂ on demand whenever the pH exceeds the desired threshold. Although the amount of CO₂ provided to the culture cannot be precisely quantified, this system allows effective pH regulation without the need for chemical buffers in the medium. This is an interesting approach, considering that, in CellDEG^®^ technology, CO₂ supply is regulated not by pH but by CO₂ consumption to maintain the desired setpoint value.

**Fig. 1:**
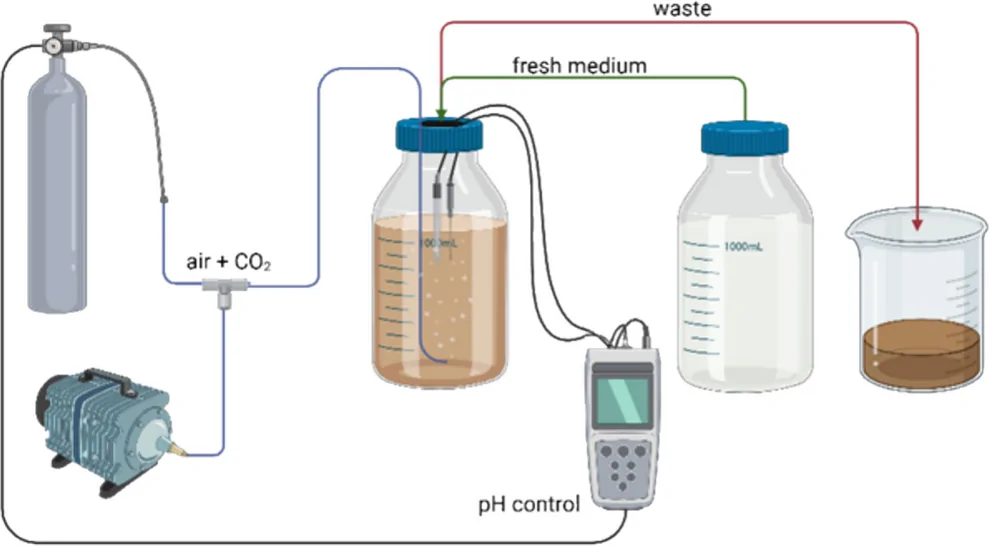
Experimental setup for the semicontinuous growth

In a semicontinuous cultivation system, the culture is diluted once daily by removing a defined volume of culture and replacing it with the same volume of fresh medium. This approach allows the maintenance of a relatively stable average concentration of nutrients and cells over time. The system was implemented in a 1-L bottle, illuminated with LEDs at an intensity of 105 µmol m⁻^2^ s⁻^1^ under continuous illumination. Several experiments were conducted to investigate the effects of residence time (*τ* = 1.8 and 2.5 days), mixing system (magnetic stirring or bubbling), and nitrogen concentration in the medium. Two nitrogen concentrations were tested: 70 mg L⁻^1^ (referred to as N70) and 140 mg L⁻^1^ (N140). Since this setup, unlike the CellDEG^®^ system, does not allow simultaneous multiple experiments, it was not possible to apply a DoE approach to the experimental plan; therefore, a one-factor-at-a-time approach was used. The experimental setup is schematized in Fig. 1 and experimental conditions are summarized in Table 3.

**Table 3:** Experimental plan with semicontinuous cultivation

Experiment	Medium	Tau (d)	Mixing
1	N70	1.8	Magnet
2	N140	1.8	Magnet
3	N140	2.5	Magnet
4	N140	2.5	Bubbling

### Analytical measurements

In CellDEG^®^ experiments, optical density (OD) at 750 nm was used for daily monitoring of the culture density (an example in Supplementary Figure S2), while the dry weight (DW) on the total biomass concentration (${m}_{tot}$, g L^−1^) was measured at the start and the end of the experiment. Such measurement allowed to determine the final ${m}_{tot}$ concentration (g L^−1^). A nitrocellulose filter with pores 0.45 µm width was firstly dried to remove humidity, by placing it in an oven at 110 °C for at least 15 min, and the filter tare was determined by a precision balance. Then, the filter was used to filtrate through a vacuum pump the ${m}_{tot}$ coming from a 5 mL sample. Then, the filtered biomass is washed with deionized water adjusted to pH 8, in order to remove residual salts while preserving the coccoliths. The filter loaded with biomass followed a drying phase in the oven for at least 2 h at, again, 110 °C. Then the exsiccated filter was weighted again on a precision balance. The ${m}_{tot}$ concentration as dry weight was then calculated as the difference between the filter loaded and tare weights.

The ${m}_{tot}$ productivity (${Pm}_{tot}$, g L^−1^ d^−1^) was calculated according to Eq. 1:

1 $${Pm}_{tot}= \frac{{C}_{X}-{C}_{X,0}}{t-{t}_{0}}$$

where C_X_ is the ${m}_{tot}$ concentration (g L^−1^) according to the dry weight and (t–t_0_) is the time interval considered, so the total batch experiment duration.

For the semicontinuous cultivation, the residence time is calculated according to Eq. 2:

2 $$\tau = \frac{{V}_{PBR}}{Q}=\frac{1}{D }$$

where V_PBR_ is the working volume of the PBR (L) and Q is the volumetric flow rate (L/h).

The determination of the concentrations of organic (m_bio,_ g L^−1^) and inorganic carbon (${m}_{carb}$, g L^−1^) in total biomass was performed using the Shimadzu TOC-LCPH instrument, equipped with TNM-L (Shimadzu Corporation, Kyoto, Japan). The analysis was performed on fresh biomass and the samples were appropriately diluted to fall within the calibration curve (5 µg L^−1^–100 mg L^−1^) before measurements. These measurements enabled the determination of ${Pm}_{tot}$ and the PIC:POC ratio achieved by the system.

In the semicontinuous experiment, samples were taken daily both from the outlet and from the freshly diluted reactor to verify that the ${m}_{tot}$ remained stable. Once a pseudo-steady concentration was reached, it was maintained for at least three days, during which samples were collected for TOC analysis and ${m}_{tot}$ measurements. In semicontinuous systems, it is not possible to achieve a true steady state, as the culture is periodically concentrated and then diluted, causing its concentration to fluctuate cyclically. However, an apparent steady state can be defined, a condition in which the average concentration remains constant over time.

### Statistical analysis

Statistical analysis was performed on biomass and coccolith productivity results obtained from semicontinuous experiments and the CellDEG^®^ experiment with calcium supplementation. In particular, Tukey’s test for multiple comparisons was performed using OriginPro 2025 at a 95% confidence level.

## Results

### Growth of *Chrysotila* sp. In high density cultivation system: a DoE approach

The effects of nitrogen concentration, light intensity, and CO₂ concentration on *Chrysotila* sp. growth under CellDEG^®^ technology resulted in values summarized in Table 4.

**Table 4:** Final ${m}_{tot}$, $P{m}_{tot}$, and PIC:POC values obtained with the CellDEG^®^ technology and DOE application

N	I	CO_2_ 1%					CO_2_ 10%				
		${m}_{tot}$	$P{m}_{tot}$	PIC/POC	POC	PIC	${m}_{tot}$	$P{m}_{tot}$	PIC/POC	POC	PIC
mg L^−1^	µmol m^−2^ s^−1^	g L^−1^	g L^−1^ d^−1^		g L^−1^	g L^−1^	g L^−1^	g L^−1^ d^−1^		g L^−1^	g L^−1^
150	150	4.42	0.63	0.088	1.619	0.142	2.80	0.4	0.018	1.302	0.023
150	375	5.14	0.74	0.054	2.099	0.113	2.26	0.32	0.016	1.059	0.017
150	600	5.32	0.76	0.072	2.046	0.147	2.74	0.39	0.019	1.267	0.024
375	150	5.07	0.72	0.058	2.042	0.118	1.17	0.17	0.031	0.516	0.016
375	375	5.97	0.85	0.043	2.537	0.108	2.27	0.32	0.018	1.053	0.019
375	600	5.68	0.81	0.038	2.448	0.094	3.22	0.46	0.015	1.519	0.022
600	150	6.00	0.86	0.041	2.562	0.105	1.15	0.16	0.057	0.466	0.027
600	375	6.71	0.96	0.044	2.832	0.126	0.744	0.11	0.051	0.307	0.016
600	600	6.59	0.94	0.044	2.784	0.122	0.513	0.07	0.074	0.196	0.015

${Pm}_{tot}$ data were analyzed using Design Expert software, which identified as the most appropriate fit a 2FI (Two-Factor Interaction) model, in which the effect of one factor on the response variable depends on the presence of another factor. The model is statistically significant (*p*-value < 0.0001) and shows a non-significant lack of fit (*p*-value > 0.05), suggesting a good match with the experimental data. The difference between the adjusted (0.711) and predicted *R*^2^ (0.681) is around 0.03, indicating a positive lack of over-fitting. The most significant parameters in the model are light and the interaction between light and CO₂, while the mutual interaction and dependence between light and nutrient and between light and CO₂ were excluded due to low significance.

For the two CO₂ conditions, the response surface of the dependent variable ${Pm}_{tot}$ to all factors tested (nutrients and light conditions), is predicted (Supplementary Figure S1) and the model for the ${m}_{tot}$ productivity retrieved as follows:

3 $${P}_{CO2 1\mathrm{\%}}=0.538891+0.00264I+0.00048N$$

4 $${P}_{CO2 10\mathrm{\%}}=0.501744+0.000264I-0.000661N$$

For the model describing the ratio between inorganic and organic carbon, the adjusted *R*^2^ (0.812) and predicted *R*^2^ (0.739) values are higher than those obtained for ${Pm}_{tot}$, and their difference (0.073) is acceptably low. Also, the model’s p-value is less than 0.0001, indicating that the model terms are statistically significant. However, the lack of fit is also significant, with a *p*-value of 0.0108. To improve the model’s performance, the experiment conducted with 600 g L^−1^ of nitrogen, light intensity of 375 µmol m^−2^ s^−1^, and 10% CO₂ was excluded from the analysis. Based on these results, the model predicts the following equations for the PIC:POC with 1% and 10% CO₂:

5 $$PIC:PO{C}_{CO2 1\mathrm{\%}}=0.113665-0.000113I-0.000188N+1.05731\times {{10}^{-7}I}^{2}+1.89578\times {10}^{-7}{N}^{2}$$

6 $$PIC:PO{C}_{CO2 10\mathrm{\%}}=0.050414-0.000113I-0.00083N+1.05731\times {{10}^{-7}I}^{2}+1.89578\times {10}^{-7}{N}^{2}$$

As shown in Table 4, the highest ${Pm}_{tot}$ are achieved under the 1% CO₂ condition. In particular, the maximum ${Pm}_{tot}$ is observed at a nitrogen concentration of 600 mg L^−1^ and a light intensity of 375 µmol m⁻^2^ s⁻^1^, reaching a value of 0.96 g L^−1^ d^−1^. The models for the ${m}_{tot}$ productivity (Eqs. 3 and 4) are suggesting an increase in $P{m}_{tot}$ with higher concentration of nutrients when the culture is supplemented with CO_2_ at 1%, while it decreases with higher concentration of nutrients under 10% of CO_2_.

Another CellDEG^®^ experiment was conducted to determine the potential effects of the photoperiod on coccolith production: no statistically significant differences are observed across all the tested conditions when compared to the results obtained in the DOE experiment, with PIC:POC values fluctuating between 0.04 and 0.06.

### CellDEG^®^ cultivation with calcium supplement

To assess a possible limitation by calcium, as a responsible factor for shell-limited production, an additional set of experiments in HCDC was carried out by increasing calcium concentration. The final ${m}_{tot}$ ranges between 4 and 6 g L^−1^ across all conditions and the highest PIC:POC ratio is obtained under the condition with 9 g L⁻^1^ of calcium chloride and 850 mg L⁻^1^ of nitrogen, reaching a final value of 0.12 ± 0.036 (Fig. 3).

To address the effect of reactor design on PIC:POC ratio, additional batch experiments were conducted in pH-controlled cylindrical reactors bubbled with air (under 150 µmol m^−2^ s^−1^), showing a productivity of 0.18 g L^−1^ d^−1^ and a final PIC:POC value of 0.17.

### Effects of semicontinuous cultivation on coccolithophores growth

To establish more favorable conditions for coccolith production, a cylindrical geometry mixed by air bubbling seems to be a good choice. However, to increase the overall productivity, semicontinuous experiments were conducted to test the influence of different nitrogen concentrations, residence times, and, in particular, mixing methods, with the aim of determining a less stressful alternative to the CellDEG^®^ technology. Analysis of total organic and inorganic carbon in the ${m}_{tot}$, obtained under apparent steady-state conditions, revealed a final PIC:POC ratio of 0.3 ± 0.093 (Table 5).

**Table 5:** Comparison of the final PIC:POC ratio obtained from semicontinuous cultivation and growth using CellDEG^®^ technology

	PIC:POC
CellDEG	0.044
N70, τ 1.8, magnet	0.198 ± 0.018
N140, τ 1.8, magnet	0.223 ± 0.037
N140, τ 2.5, magnet	0.216 ± 0.035
N140, τ 2.5, bubbling	0.302 ± 0.093

With this setup, the final $P{m}_{tot}$ value (Fig. 4) was 0.27 g L^−1^ d^−1^.

## Discussion

### Biomass productivity and coccolith production in the CellDEG^®^ system with a DoE approach

In this work, the biomass productivity of *C. carterae* was assessed as a function of different variables, showing the highest ${Pm}_{tot}$ (0.96 g L^−1^ d^−1^) with 1% CO₂, 600 mg L^−1^ of nitrogen and a light intensity of 375 µmol m⁻^2^ s⁻^1^. However, it should also be noted that, although productivity increases with light intensity, photon utilization efficiency decreases due to possible saturation phenomena, which is a common feature of photosynthetic microorganisms (Sforza et al. 2015). Nevertheless, the productivity values obtained in this study are generally much higher than those reported in the literature for *C. carterae*, such as the 0.56 g L^−1^ d^−1^ (Moheimani et al. 2011) or the 0.17 g L^−1^ d^−1^ (Dolganyuk et al. 2023), indicating that an HCDC system such as the CellDEG^®^ technology can support higher biomass proliferation with this species than a traditional batch cultivation system.

Some interactions among factors were observed, as biomass productivity is boosted under higher concentration of nutrients and CO_2_ at 1%, while it decreases with higher concentration of nutrients under 10% of CO_2_. In this condition, it is likely that a CO₂ inhibition effect occurred, similar to what was reported for *Gephyrocapsa huxleyi* in the study by Segovia et al. (Segovia et al. 2017), where elevated CO₂ supply led to a reduction in biomass growth, from 1600 to 200 µg_C_ L^−1^.

The results in terms of particulate inorganic carbon (PIC), particulate organic carbon (POC), and their ratio (Table 4) show a significantly different trend from that usually reported for this species. Based on the PIC:POC ratio obtained, it can be observed that the results under all tested conditions are significantly lower than the value reported in the literature for *Chrysotila* species, which ranges from 0.17 to 0.8 (Gafar et al. 2019; Zou et al. 2017). The highest PIC:POC value obtained in this experiment is only 0.074, suggesting that the production of the cell’s calcium carbonate shell under these conditions was minimal.

**Fig. 2:**
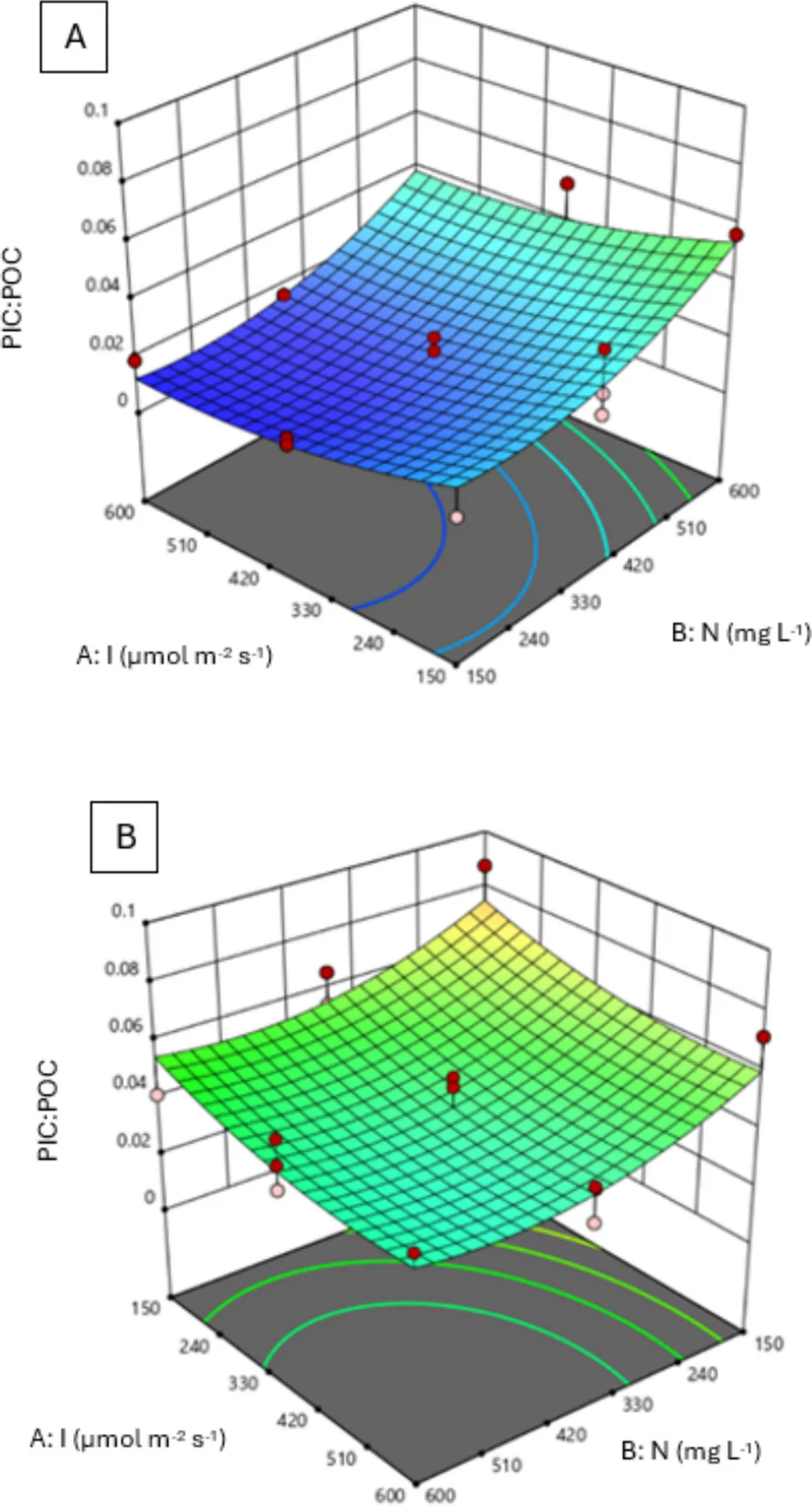
Graphical representations of the PIC:POC ratio models with the two CO_2_ conditions: 1% (**A**) and 10% (**B**). For both conditions, a decrease in the ratio is observed with increasing nutrient concentration and light intensity

The PIC:POC ratio, (Fig. 2) decreased with increasing light intensity and nutrient concentration, but exposing the culture to periods of darkness did not influence coccolith production. This finding reinforces the conclusions drawn by several studies indicating that the ability of coccolithophores to produce calcium carbonate is significantly reduced under dark conditions, with some studies reporting reduced production (Bretherton et al. 2019; Paasche 1966) and others even observing a complete absence of it (Maltsev et al. 2021). As such, the photoperiod can be excluded as a potential contributing factor to the low results observed in the previous experiment. Other factors may account for the reduced production, particularly the concentration of calcium in the culture medium: coccoliths are composed of calcium carbonate, meaning that calcium is essential for their synthesis. However, the calcium concentration in the medium, derived from the sea salts, resembles that of natural seawater, approximately 400 mg L^−1^ (Palandri et al. 2026). Given the 1:1 stoichiometric ratio with calcium carbonate, the theoretical maximum PIC concentration would be around 120 mg L^−1^. This aligns closely with the highest PIC value obtained in this experiment, which was around 147 mg L^−1^, slightly higher than the calculated theoretical maximum. Thus, as the ${m}_{tot}$ in this system reached an unexpected very high concentration (which is however typical for other species in such a cultivation system), a possible Ca limitation could have been occurred. No previous studies have specifically investigated the amount of calcium required for coccolithophore calcification in high density cultivation, although calcium toxicity has been examined in several studies involving different species, such as *Gephyrocapsa huxleyi,* when exposed to Ca concentrations higher than 50 mmol L⁻^1^ (Müller et al. 2011). In this work, *Chrysotila* sp. appears to be unaffected by calcium levels up to 200 mmol L⁻^1^. Another possible hypothesis on poor coccoliths production is the effect of CO₂ accumulation in the medium and the resulting decrease in pH. Indeed, an equilibrium exists between bicarbonate, carbonate, and CO₂ that is primarily governed by pH. If the pH goes down below 6.35, the predominant inorganic form is CO₂ (Ghobadi et al. 2016). Therefore, acidification may facilitate the conversion of calcium carbonate within coccoliths into CO₂, which is subsequently removed from the medium via the CellDEG^®^ aeration system. This chemical behavior under low-pH conditions was also assessed by Hassenkam et al. (2011) in their study of *Coccolithus pelagicus*, confirmed in other species (Kottmeier et al. 2022; Bach et al. 2011). However, measurements of the culture pH at the end of the experiment showed a final value of around 8 (not shown), ruling out pH as a possible cause. The method of mixing may also influence coccolith production. The CellDEG^®^ technology employs orbital shaking platforms to maintain culture homogeneity. Some studies conducted on *C. carterae* have shown that this genus is particularly sensitive to mixing conditions and shear stress (Moheimani et al. 2011), suggesting that similar responses may occur in other *Chrysotila* species, potentially making this technology unsuitable for the genus.

### CellDEG^®^ with calcium supplement alone is insufficient to significantly enhance coccolith production

**Fig. 3:**
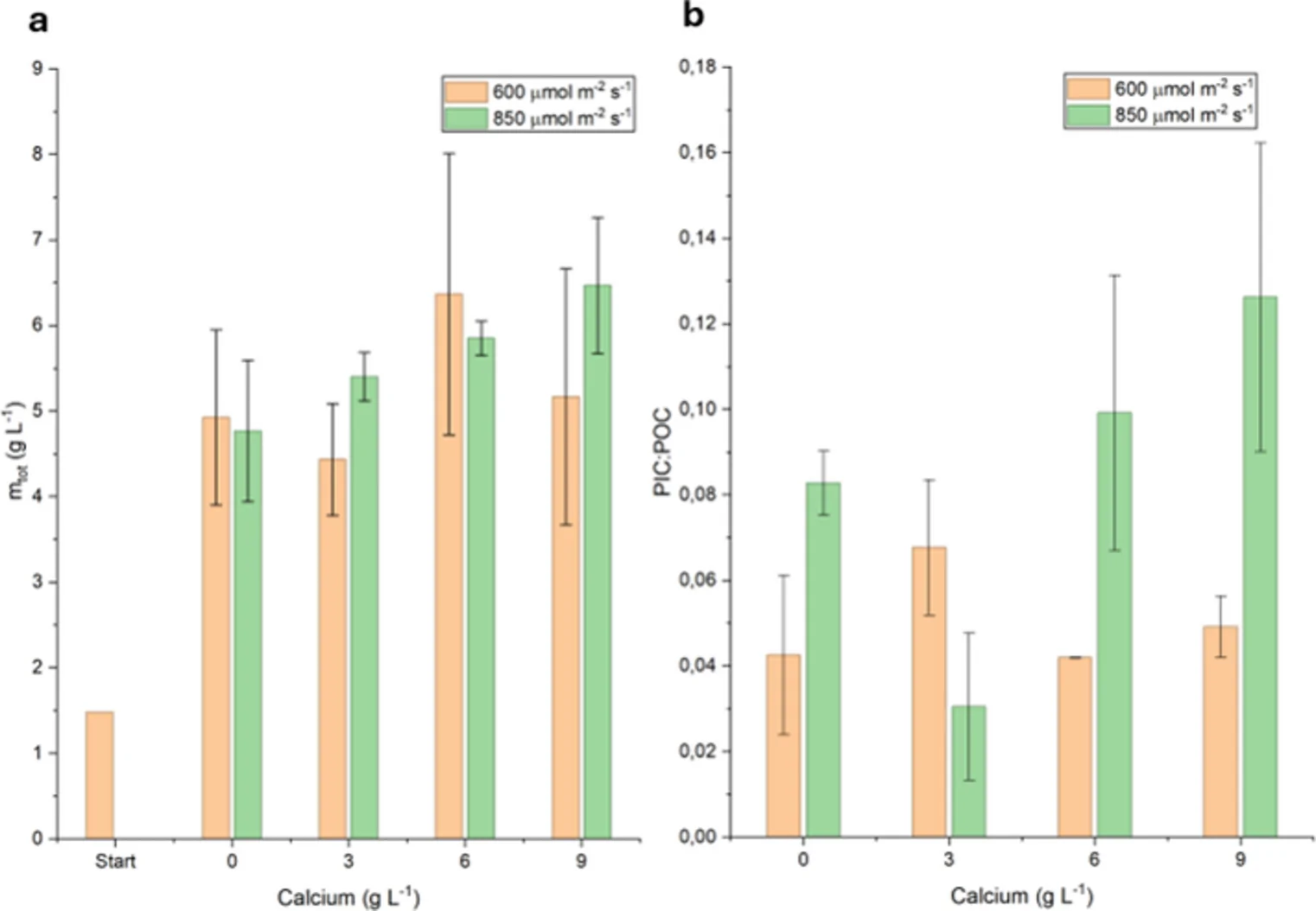
Final ${m}_{tot}$ (**A**) and PIC:POC ratio (**B**) obtained at two nitrogen concentrations of 600 and 800 mg L⁻^1^ (milligrams of nitrogen per liter), with different calcium chloride supplementation (0, 3, 6, 9 g L^−1^) using the CellDEG^®^ technology. The bars represent the standard deviation, and the letters indicate groups of results that are statistically different from each other

For what regard the coccolith productivity, the final ${m}_{tot}$ obtained supplementing calcium in CellDEG^®^ is overall comparable to those of the experiments without Ca supplementation (6.71 g L^−1^ in the best conditions). Considering the condition with the highest ${m}_{tot}$ (6.46 g L^−1^ from the condition with 9 g L^−1^ of calcium chloride and 850 mg L^−1^ of nitrogen) and assuming, hypothetically, that this value includes only the m_bio_ and not the ${m}_{carb}$ (as it actually does), the amount of calcium required for coccolith formation, based on a PIC:POC ratio of 0.17 would be approximately 1.7 g L^−1^. Since this is lower than the amount of calcium provided under that specific condition, there should be more than enough calcium available for coccolith formation. By contrast, the results obtained (Fig. 3) show that the conditions with 600 mg L^−1^ of nitrogen do not exhibit any improvement in the PIC:POC ratio compared to those obtained in the DoE-based experiment or the control condition, regardless of the calcium concentration supplied. Since the optimal ${m}_{tot}$ calcium on coccolith production. Consistently, an improvement was observed under conditions with higher nutrient availability (850 mg L^−1^ of nitrogen), particularly when higher amounts of calcium chloride are provided. However, the desired PIC:POC values are still not achieved. In fact, the highest ratio obtained (0.12) is still lower than the expected value of 0.17. This suggests that calcium may be one of the limiting factors for coccolith productivity, as also demonstrated by other studies, such as those by Langer et al. (2007) on *Emiliania huxleyi* it is clearly not the only factor controlling coccolith production. Other factors may have affected coccolith synthesis or stability, particularly the mixing technology, as previously hypothesized. Since these potential factors were not accounted for in the model, they may also explain the significant lack of fit observed. Thus, in summary, it appears that high density cultivation systems, based on an increased CO_2_ transfer, is actually very effective in boosting the production of coccolithophores, more than ever obtained in other systems, but at the expenses of the shell production.

The additional batch experiments conducted in pH-controlled cylindrical reactors showed a lower productivity than the corresponding value obtained in CellDEG^®^, but a much higher final PIC:POC ratio, suggesting that to boost coccolith production, the reactor design, including the mixing, is strongly relevant.

### Advantages of the semicontinuous cultivation on the coccolith production

While variations in nitrogen concentration and residence time did not result in significant differences, the experiment utilizing a bubbling mixing system (as opposed to magnetic stirring) produced the most favorable outcome in terms of the PIC:POC ratio. Although no studies currently describe the effect of mixing on coccolith production, numerous studies highlight the importance of mixing conditions for metabolite production in microalgal systems (Ghosh et al. 2024; Wu et al. 2024). The value obtained (0.3) is not only higher than that observed in cultures grown using CellDEG^®^ technology (Table 5), but it is also nearly two times the average PIC:POC ratio reported in the literature for *C. carterae* by Gafar et al. (2019) (0.17). These findings suggest that light bubbling as a mixing method may be less stressful than the orbital shaking used in the CellDEG^®^ system.

**Fig. 4:**
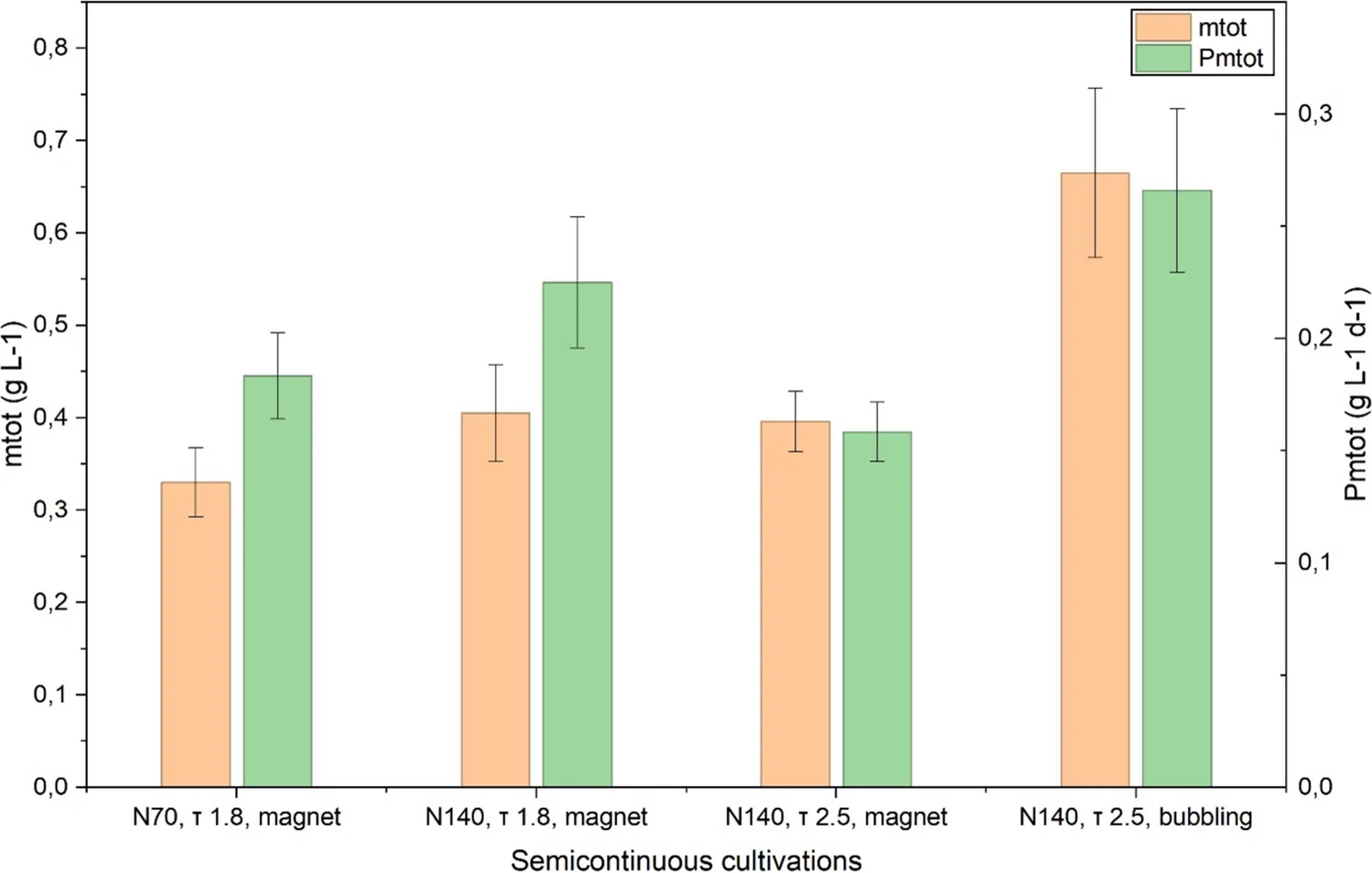
Comparison of the final ${m}_{tot}$ and $P{m}_{tot}$ obtained from semicontinuous cultivation and growth using CellDEG^®^ technology. The bars represent the standard deviation, and the letters indicate groups of results that are statistically different from each other

However, the ${Pm}_{tot}$ (Fig. 4) remained significantly lower than that achieved using CellDEG^®^ technology. The final value (0.27 g L^−1^ d^−1^) was slightly more than one-third of the productivity obtained with CellDEG^®^ (0.96 g L^−1^ d^−1^). Anyway, this value of productivity was much higher than the one obtained in batch in the same reactor geometry. Thus, if the production is focused to the commercialization of coccoliths, it would likely find it more advantageous to optimize ${m}_{tot}$ production using semicontinuous cultivation. Conversely, an industrial process primarily oriented toward ${m}_{tot}$ production and commercialization (rather than coccolith yield) would find the CellDEG^®^ technology significantly more suitable for their needs.

## Conclusions

This study, in addition to representing the first application of this technology to coccolithophore species, demonstrated the high potential of the CellDEG^®^ technology, a high cell density culture system, for biomass production, achieving a final yield of 0.96 g L^−1^ d^−1^ at a nitrogen concentration of 600 mg L^−1^ and a light intensity of 375 µmol m^−2^ s^−1^. These results highlight the promise of this technology for industrial applications focused on the production and commercialization of microalgal biomass. On the other hand, coccolith production was found to be very limited in such a system. Experiments conducted using this technology demonstrate that the limitation in coccolith production is not due to the light–dark photoperiod, and that calcium concentration alone is not the only contributing factor, while the orbital shaking could be the responsible of such a result, as the genus *Chrysotyla* is susceptible to the mixing. Studies conducted under semicontinuous growth with light bubbling mixing reported a final PIC:POC ratio of 0.3, nearly twice the average value found in the literature (0.17). This suggests that such an approach may be optimal for industrial applications targeting coccoliths as the main product, even though reducing the overall mass productivity. In this research, maintaining an optimal medium pH through controlled CO₂ release proved to be a suboptimal solution, since, if the potential application of this species is carbon capture from flue gases, CO₂ supply cannot be restricted. Furthermore, the bubbling-based mixing system required constant maintenance due to reactor fouling and cleaning needs. Therefore, future studies should focus on further optimizing continuous cultivation, while also identifying more efficient strategies for pH control and developing improved bubbling technologies.

## Supplementary Information

Below is the link to the electronic supplementary material.

ESM 1 Supplementary Material 1 (DOCX 237 KB)

## Data Availability

Data will be made available on request. The supplementary data of this word can be found in online version of the paper.
