# Supplementary material for: Biomass and coccolith productions in Chrysotila sp. in high density cultivation system
Source: Appl Microbiol Biotechnol. 2026 Jun 10;110:251. doi: 10.1007/s00253-026-13907-w (PMC13478033; doi:10.1007/s00253-026-13907-w)
Supplement: Supplementary file 1 [file 253_2026_13907_MOESM1_ESM.docx]

**Biomass and coccolith productions in *Chrysotila carterae* in high density cultivation system**

Matteo Ferrari^a^, Veronica Buson^a^, Elisa Palandri^a^, Manuela Bordiga^b^, Adriano Carrara *^c^,* Eleonora Sforza^a^,

*^a^Department of Industrial Engineering DII, University of Padova, Via Marzolo 9, 35131 Padova, Italy*

*^b^ National Institute of Oceanography and Applied Geophysics (OGS), Borgo Grotta Gigante 42/C,*

*34010 Sgonico ( TS ), Italy*

*^c^ A2A S.p.A., Brescia, Italy*

Supplementary Material

Submitted to Applied microbiology and biotechnology

April 2026

^*^Corresponding author: Eleonora Sforza

e-mail address: [eleonora.sforza@unipd.it](mailto:eleonora.sforza@unipd.it)

 a)

B: Nitrogen (mg L^-1^)

B: Nitrogen (mg L^-1^)

Biomass productivity (g L^-1^ d^-1^)

A: Light intensity (µmol m⁻² s⁻¹)

A: Light intensity (µmol m⁻² s⁻¹)

Biomass productivity (g L^-1^ d^-1^)

b)

Figure S1: graphical representations of the biomass productivity models with the two CO_2_ conditions: 1% (a) and 10% (b).

Figure S2: An example of the temporal trend of optical density (OD) observed during a CellDEG experiment with coccolithophores. In this case, daily OD measurements were collected for all tested conditions supplemented with different calcium concentrations.
